# Effectiveness of blended learning versus conventional didactic teaching in pathology education outcomes

**DOI:** 10.6026/973206300223781

**Published:** 2026-06-30

**Authors:** Puja Singh, Jyoti Namdev, Jyoti Shukla

**Affiliations:** 1 Department of Pathology, Bundelkhand Medical College, Sagar, Madhya Pradesh, India;

**Keywords:** Blended learning, teaching-learning methods, medical education, learner engagement and competency-based medical education (CBME)

## Abstract

Traditional didactic lectures in pathology often promote passive learning, limiting students' ability to apply knowledge clinically. Therefore, it is of interest to compare the learner outcomes between blended learning and traditional didactic lectures among 110 second-year MBBS students. The blended learning group demonstrated significantly superior performance in application-based scores (mean difference 4.5, p<0.001, d=1.85), total examination scores (difference 5.5, p<0.001, d=1.48) and all learner engagement domains (p<0.001) with large effect sizes. Student satisfaction was also markedly higher with blended learning (4.32±0.51 versus 3.48±0.62, p<0.001). Thus, data shows the blended learning enhances higher-order cognitive skills, engagement and satisfaction into competency-based medical curricula for pathology education.

## Background:

For decades, the didactic lecture has been the cornerstone of medical education, yet its limitations are hard to ignore [1, 2, 3]. Students often sit passively, their engagement waning within minutes and retention of visually and conceptually dense discipline like pathology suffers as a result [4, 5]. Active learning strategies improve knowledge acquisition, but translating that into crowded pre-clinical curricula remains challenging [3, 6 and 7]. Blended learning-which combines online digital media with traditional face-to-face teaching offers a promising alternative [8, 9, 10]. It allows students to control the pace of foundational content delivery, freeing up in-person time for application and discussion [8, 9 and 11]. Therefore, it is of interest to compare the blended and traditional teaching methods in pathology education remains limited.

## Methodology:

This comparative, quasi-experimental and prospective study was conducted at Bundelkhand Government Medical College, Sagar, India, for duration of 6 months from Oct 2025. The study aimed to evaluate and compare the effectiveness of blended learning and traditional didactic lectures on learner outcomes among undergraduate medical students studying pathology. Due ethical approval taken from institute ethical committee and written informed consent was obtained from all participants. A total of 120 second-year MBBS students were included and allocated into two groups using simple randomization. Group A received conventional didactic lectures, while Group B underwent blended learning sessions. Exclusion criteria included prior pathology coursework (n = 3) and failure to attend more than 80% of sessions (n = 7), leaving 110 students for final analysis. The traditional teaching group attended faculty-led classroom lectures delivered using Powerpoint presentations and chalk-and-board explanations. Each session lasted approximately one hour and followed the routine departmental teaching schedule. In contrast, the blended learning group was exposed to a combination of face-to-face teaching, online learning resources, case-based discussions, flipped classrooms and self-directed learning modules. Digital content included histopathology images, quizzes and supplementary reading material shared via Whatsapp group. Interactive discussions and formative assessments were incorporated to encourage active participation and reflective learning. Both groups were taught the same pathology topics by the same faculty members to minimize instructor-related variation. The duration of teaching, learning objectives and assessment patterns were kept uniform across groups. Performance was measured using a 40-item multiple-choice examination administered one week after the intervention. Questionnaire had, prepared and validated by subject experts in pathology and medical education, Cronbach's alpha = 0.82 and covered both recall and clinical application. Prior to the intervention, all students completed a pre-test consisting of multiple-choice questions designed to assess baseline knowledge. At the end of the teaching sessions, a post-test of similar difficulty and content coverage was administered to evaluate immediate learning outcomes. Engagement was assessed using two approaches. First, we adapted the 12-item Student Course Engagement Questionnaire (SCEQ), previously validated in medical education settings, measuring behavioural (attendance, attention), emotional (interest, enthusiasm) and cognitive (self-regulation, deep learning) domains. Second, we recorded attendance rates and, for the blended group, participation on quizzes etc. In addition to cognitive assessment, student perceptions regarding teaching effectiveness, engagement and satisfaction were collected using a structured feedback questionnaire based on a five-point Likert scale. The questionnaire explored domains such as clarity of concepts, interaction during sessions, motivation for self-learning and overall learning experience. Reliability of the feedback tool was assessed using Cronbach's alpha.

## Statistical analysis:

Statistical analysis was performed using Python (v3.9), Pandas (v1.3.5) and Seaborn (v0.11.2). Pre-test and post-test scores within groups were compared using paired t-test, while intergroup comparisons were performed using independent t-test. A p-value < 0.05 was considered statistically significant.

## Results:

A total of 110 second-year MBBS students participated in the study, with 55 students each in the traditional didactic lecture group (Group A) and the blended learning group (Group B). Baseline demographic characteristics and pre-test scores were comparable between the two groups, indicating homogeneity at the beginning of the intervention. In this comparative study, baseline characteristics were similar between groups (Table 1). Group A (didactic lecture, n=55) had a mean age of 18.46 ± 1.29 years and a male-to-female ratio of 33:22. Group B (blended learning, n=55) had a mean age of 18.23 ± 1.34 years and a ratio of 35:20. Mean pre-test scores were comparable: 11.84 ± 2.31 (Group A) versus 12.02 ± 2.45 (Group B). All between-group differences were non-significant (p > 0.05), confirming randomization success and establishing a valid baseline for post-intervention comparisons. Table 2 demonstrates that blended learning significantly outperformed traditional lectures across all academic outcomes. While recall scores improved modestly (mean difference 1.0, 95% CI 0.3-1.7; p=0.008; d=0.52), application skills showed a large effect (difference 4.5, 95% CI 3.6-5.4; p<0.001; d=1.85). Total examination scores favoured blended learning substantially (difference 5.5, 95% CI 4.1-6.9; p<0.001; d=1.48), indicating superior overall learner performance. The Table 3 shows significantly higher learner outcomes in the blended learning group across all domains. Mean differences favoured Group B: behavioural (2.8, 95% CI 2.0-3.6; p<0.001; d=1.37), emotional (3.4, 95% CI 2.5-4.3; p<0.001; d=1.50) and cognitive (3.1, 95% CI 2.2-4.0; p<0.001; d=1.28). Overall scores were markedly higher with blended learning (48.6±5.2 vs. 39.3±6.1; difference 9.3, 95% CI 7.1-11.5; p<0.001; d=1.62), indicating large educational benefits. The Table 4 presents learner satisfaction outcomes favouring blended learning (Group B) over didactic lectures (Group A). Group B demonstrated significantly higher scores in conceptual clarity (1.34±0.14 vs. 1.01±0.24, p<0.05), student engagement (1.72±0.17 vs. 1.28±0.15, p<0.001) and motivation for self-learning (1.32±0.20 vs. 1.19±0.23, p<0.001). Overall satisfaction was notably greater with blended learning (4.32±0.51 vs. 3.48±0.62, p<0.001), indicating enhanced learner perceptions of active learning methods. Reliability analysis of the feedback instrument showed good internal consistency with a Cronbach's alpha value greater than 0.80. Overall, the findings suggest that while conventional lectures remain useful for structured content delivery, blended learning provides a more engaging and effective educational environment that positively influences learner performance and satisfaction in undergraduate pathology education.

## Discussion:

The present study demonstrates that blended learning significantly outperforms traditional didactic lectures in undergraduate pathology education, particularly in application-based knowledge (mean difference 4.5, p<0.001, d=1.85) and learner engagement domains (behavioural: d=1.37; emotional: d=1.50). While recall scores improved modestly with blended learning (d=0.52), the large effect sizes for higher-order cognitive skills underscore the pedagogical advantage of active, multimodal learning strategies in medical education. Our finding that blended learning enhances application skills aligns closely with previous studies comparing active learning methodologies to traditional lectures. Similar to the observations of Naik *et al.*, who reported that case-based learning produced superior performance in application-based and critical thinking questions; our results confirm that blended approaches foster deeper cognitive processing [1]. Likewise, Thejaswini *et al.* demonstrated that team-based learning yielded a 24% improvement in higher-order thinking questions compared to didactic lectures, a finding consistent with our large effect sizes for application sub-scores [6]. Singh *et al.* further corroborated these results, showing that integrated video-based learning produced significantly higher post-test scores than conventional teaching across multi-centric settings [12]. In contrast to our findings, some studies have reported comparable overall academic performance between active and passive learning methods. Kashif *et al.* found that while blended learning produced the highest post-test scores among three teaching modalities, overall knowledge acquisition was not significantly different between e-learning and didactic lectures [5]. Similarly, Liu *et al.* observed that blended learning significantly enhanced knowledge acquisition (change scores 4.05 versus 2.00, p=0.022), consistent with our results, though their effect sizes were more modest [8]. The systematic review by Maia *et al.* provides broader context, reporting large effects for CBL on exam scores (SMD=2.37) but noting very low certainty of evidence due to methodological heterogeneity - a caveat relevant to our study as well [3]. The substantial improvement in learner engagement and satisfaction observed in our study (overall satisfaction: 4.32±0.51 vs. 3.48±0.62, p<0.001) is consistent with multiple earlier reports. Jain *et al.* [7] documented that 82.21% of students agreed that case-based learning improved understanding, while Nishal *et al.* [13] reported 87% student endorsement of CBL for better topic retention. Zhong *et al.* [9] similarly found that blended learning students rated online learning satisfaction highly (4.03±0.67) and demonstrated correlation between online components and overall satisfaction (r=0.581, p<0.05). Notably, our Cronbach's alpha (>0.80) confirms the reliability of our feedback instrument, strengthening confidence in these perceptual findings. The biological plausibility of our findings rests on cognitive load theory and constructivist learning principles. Blended learning reduces extraneous cognitive load by allowing self-paced engagement with foundational content before classroom application, freeing working memory for higher-order processing during face-to-face interactions. As per study by Pranathi *et al.* [11], blended group excelled in application while maintaining recall competency. The flipped classroom approach described by Zhong *et al.* [9] similarly demonstrated that physical classroom interaction following online preparation enhanced knowledge construction compared to virtual-only formats. Several strengths of this study warrant mention. The crossover design for baseline equivalence ensured comparability between groups, as evidenced by non-significant differences in age, gender distribution and pre-test scores. The use of validated assessment instruments with demonstrated internal consistency (Cronbach's alpha >0.80) enhances measurement reliability. Furthermore, the inclusion of both cognitive (recall and application sub-scores) and affective (engagement domains and satisfaction) outcomes provides a multidimensional evaluation of educational effectiveness. However, several limitations must be acknowledged. The single-centre design at a tertiary care hospital may limit generalisability of the findings. The sample size of 110 has insufficient power for smaller but educationally meaningful differences. Short-term assessment cannot address knowledge retention over time, a critical outcome for medical education where longitudinal competency is paramount. Potential confounding variables not controlled include prior academic achievement, baseline technological literacy and individual learning style preferences, all of which may influence responsiveness to blended learning. Additionally, the absence of blinding (inherent to educational interventions) introduces potential performance and detection bias, though our use of standardised assessments mitigates this concern. In conclusion, the present study provides robust evidence that blended learning enhances application-based knowledge and learner engagement compared to traditional didactic lectures in undergraduate pathology education. These findings support the integration of active, multimodal teaching strategies into medical curricula, aligning with competency-based medical education frameworks that emphasise higher-order cognitive skills and self-directed learning.

## Conclusion:

Blended learning significantly outperformed traditional didactic lectures in enhancing application-based knowledge, learner engagement and overall satisfaction in undergraduate pathology education. While recall scores improved modestly, the large effect sizes for higher-order cognitive skills support integrating active, multimodal strategies into medical curricula to foster deeper learning and clinical reasoning.

## Figures and Tables

**Table 1:** Baseline characteristics of study participants

**Variable**	**Group A (Didactic Lecture)**	**Group B (Blended Learning)**	**p-Value**
Mean age (years)	18.46 ± 1.29	18.23 ± 1.34	> 0.05
Gender (M/F)	33/22	35/20	> 0.05
Mean Pre-Test Score	11.84 ± 2.31	12.02 ± 2.45	> 0.05

**Table 2:** Comparison of learner academic outcomes between blended learning and didactic lecture groups

**Outcome measure**	**Group A (Didactic Lecture)**	**Group B (Blended Learning)**	**Mean difference (95% CI)**	**p-value**	**Effect size (Cohen's d)**
Recall sub-score (out of 20)	17.4 ± 2.0	18.4 ± 1.8	1.0 (0.3 to 1.7)	0.008	0.52
Application sub-score (out of 20)	11.3 ± 2.6	15.8 ± 2.1	4.5 (3.6 to 5.4)	< 0.001	1.85
Total examination score (out of 40)	28.7 ± 4.2	34.2 ± 3.1	5.5 (4.1 to 6.9)	< 0.001	1.48

**Table 3:** Comparison of Learner Engagement between blended learning and didactic lecture groups

**Outcome measure**	**Group A**	**Group B (Blended Learning)**	**Mean difference (95% CI)**	**p-value**	**Effect size (Cohen's d)**
Behavioural domain (out of 20)	12.1 ± 2.2	14.9 ± 1.8	2.8 (2.0 to 3.6)	< 0.001	1.37
Emotional domain (out of 20)	12.8 ± 2.4	16.2 ± 2.0	3.4 (2.5 to 4.3)	< 0.001	1.5
Cognitive domain (out of 20)	14.4 ± 2.5	17.5 ± 2.3	3.1 (2.2 to 4.0)	< 0.001	1.28
Overall Score (out of 60)	39.3 ± 6.1	48.6 ± 5.2	9.3 (7.1 to 11.5)	< 0.001	1.62

**Table 4:** Student perception and satisfaction scores based on student feedback

**Variable**	**Group A (Didactic Lecture)**	**Group B (Blended Learning)**	**p-Value**
Conceptual Clarity	1.01 ± 0.24	1.34 ± 0.14	<0.05
Student Engagement	1.28 ± 0.15	1.72 ± 0.17	<0.001
Motivation for Self-Learning	1.19 ± 0.23	1.32 ± 0.20	<0.001
Overall Satisfaction	3.48 ± 0.62	4.32 ± 0.51	<0.001
